# Duvelisib: A 2018 Novel FDA-Approved Small Molecule Inhibiting Phosphoinositide 3-Kinases

**DOI:** 10.3390/ph12020069

**Published:** 2019-05-06

**Authors:** Daniel A. Rodrigues, Fernanda S. Sagrillo, Carlos A. M. Fraga

**Affiliations:** 1 Laboratório de Avaliação e Síntese de Substâncias Bioativas (LASSBio), Instituto de Ciências Biomédicas, Universidade Federal do Rio de Janeiro, PO Box 68023, Rio de Janeiro, RJ 21941-902, Brazil; dar.go.qfm@gmail.com (D.A.R.); fefa_uff@yahoo.com.br (F.S.S.); 2 Programa de Pós-Graduação em Química, Instituto de Química, Universidade Federal do Rio de Janeiro, Rio de Janeiro, RJ 21941-909, Brazil

**Keywords:** approved drugs, duvelisib, Copiktra, FDA, phosphoinositide 3-kinases, cancer

## Abstract

Duvelisib (Copiktra^®^) is a dual inhibitor of phosphoinositide 3-kinases (PI3Kδ and PI3Kγ). In 2018, duvelisib was first approved by the Food and Drug Administration (FDA) for the treatment of adult patients with relapsed or refractory chronic lymphocytic leukaemia (CLL)/ small lymphocytic lymphoma (SLL) after at least two prior therapies. Duvelisib has also been approved under accelerated track for relapsed or refractory follicular lymphoma (FL) after at least two prior systemic therapies. In this review, we provide a series of information about duvelisib, such as the development of clinical trials for LLC/SLL and FL and the steps used for its synthesis.

## 1. Introduction

**Figure 1:**
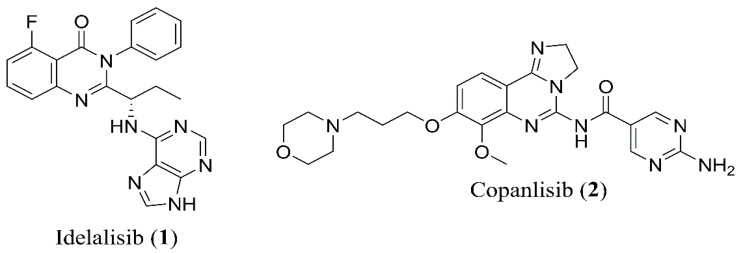
Chemical structure of PI3K inhibitor drugs idelalisib (**1**) and copanlisib (**2**).

In 2014, Food and Drug Administration (FDA) approved the idelalisib (**1**), a first-in-class inhibitor of PI3Kδ, for the treatment of relapsed chronic lymphocytic leukaemia (CLL), relapsed follicular B-cell non-Hodgkin lymphoma (NHL) and relapsed small lymphocytic leukaemia (SLL) [1]. Although idelalisib (**1**) (Figure 1) possesses an enhanced selectivity for the PI3Kδ, it opened a new road ahead for the family of phosphoinositide 3-kinase (PI3K) inhibitors. In 2017, the approval of copanlisib (**2**) (Figure 1) for the treatment of relapsed follicular lymphoma (FL) and the ongoing trials on several hematological and solid malignancies has showed the usefulness of the pan-PI3K inhibitors in the clinics [2,3].

The purpose of this review is to provide a series of information on the third PI3K inhibitor approved by FDA in 2018: duvelisib (IPI-145, formerly developed by Intellikine and further developed by Infinity Pharmaceuticals, which granted to Verastem Oncology in 2016 the rights of development and commercialization worldwide [4]).

## 2. Duvelisib

### 2.1. Names and Structure

**Figure 2:**
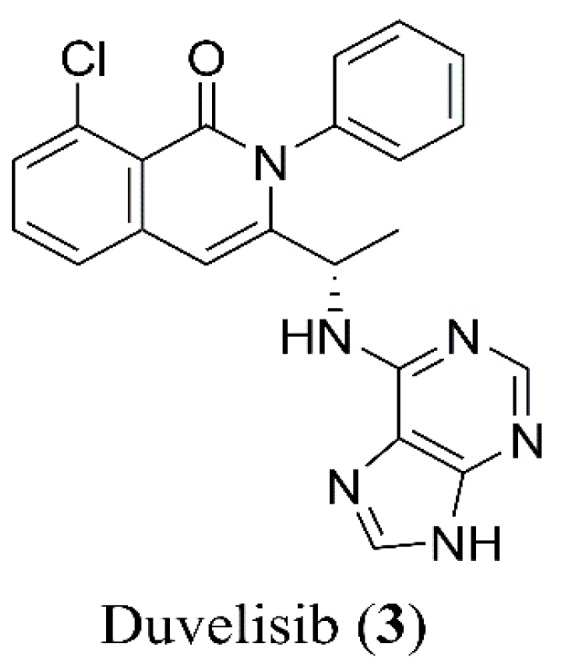
Chemical structure of duvelisib (**3**).

Duvelisib (**3**, Figure 2) is commercialized by Verastem Oncology, under the brand name Copiktra^®^. Its IUPAC name is: (*S*)-3-(1-((*7H*-purin-6-yl)amino)ethyl)-8-chloro-2-phenylisoquinolin-1(*2H*)-one, CAS 1201438-56-3.

### 2.2. Uses

Duvelisib (**3**) was first approved on September 24, 2018, in the USA, for the treatment of adult patients with relapsed or refractory CLL/ SLL after at least two prior therapies (NCT02004522) [5,6]. Based on overall response rate (ORR) the drug has also been approved under fast track for the treatment of relapsed or refractory FL after at least two prior systemic therapies, but the continued approval depends on the clinical benefit demonstrated in the trials (NCT02204982) [7].

### 2.3. Targets and Selectivity

The phosphoinositide 3-kinases (PI3Ks) play an important role in various processes, such as proliferation, differentiation, motility, survival, and intracellular trafficking [8,9,10,11]. This signaling pathway is commonly activated in cancer [10]. There are three classes of PI3K, but the drug discovery process is mainly focused in the enzymes of class I, which is divided into class IA (PI3Kα, PI3Kβ and PI3Kδ) and IB (PI3Kγ) [12].

Duvelisib (**3**) is a dual PI3Kδ and PI3Kγ inhibitor with enhanced selectivity for PI3Kδ (10-fold) over PI3Kγ (Table 1). The selectivity for PI3Kδ over PI3Kα can be attributed to the conformation that the compounds adopt in the active site, namely propeller-shaped, which opens a hydrophobic pocket that is not present in the apoenzyme [13,14]. Duvelisib (**3**) was shown to be selective for the inhibition of PI3K from class I, not being active against other protein or lipid kinases [15].

**Figure 3:**
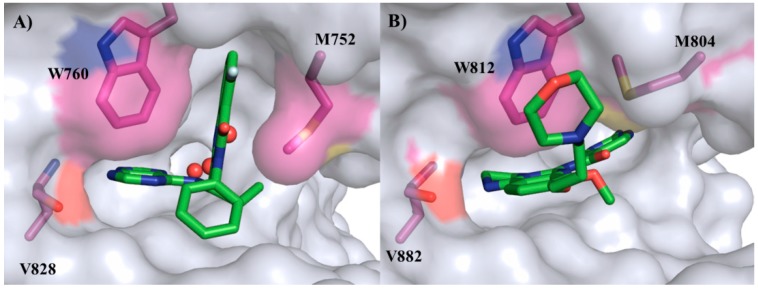
Binding modes of idelalisib (**1**) in PI3Kδ (PDB:4XE0) (**A**) and copanlisib (**2**) in PI3Kγ (PDB:5G2N) (**B**).

Although there is no crystal structure of the duvelisib (**3**) in the active site of PI3K, this drug should possess a similar interaction mode of idelalisib (**1**), given the structural similarities between these two compounds. Figure 3 presents the binding modes of idelalisib (**1**) in PI3Kδ (PDB:4XE0) [13] and copanlisib (**2**) in PI3Kγ (PDB:5G2N) [3].

**Figure 4:**
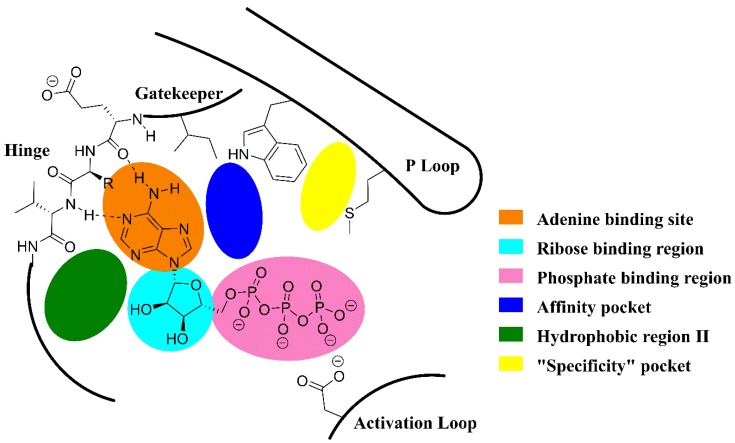
General model for the design of PI3K inhibitors [18,19].

The duvelisib (**3**) binds at the ATP binding region, which is one of the most conserved throughout the PI3K family [8,16,17]. It is possible to explore the model generally used for the development of protein kinase inhibitors, incorporating features related to the inhibition of PI3K [18,19]. In general, PI3K inhibitors form hydrogen bond interactions in the region of the hinge, occupying the adenine binding site (Figure 4). The presence of the hydrophobic regions I and II in the active site of PI3K should be noted [20]. The former is also called the “affinity pocket” and it is commonly exploited by pan-PI3K inhibitors through a series of polar interactions. On the other hand, the hydrophobic region II is a region exposed to the solvent [20]. The compounds with the so-called propeller-shaped conformation open a hydrophobic pocket between the residues of tryptophan and methionine, also known as the “specificity pocket”, conferring selectivity for PI3Kδ [20] (Figure 4).

Although the duvelisib (**3**) presents a high similarity to idelalisib (**1**), it possesses unique features related with to the binding affinity by PI3K. Duvelisib (**3**) presents a long target residence time, which may be closely associated with more durable effects [15].

### 2.4. Clinical Trials

The differential profile of the duvelisib (**3**) is based on the dual inhibition of PI3Kδ and PI3Kγ, which are mainly expressed in the immune system [10,21]. They present a non-overlapping cellular function in the immune system, considering the innate and adaptive immune response [22,23,24]. Several clinical trials with duvelisib (**3**) are completed and ongoing for the treatment of cancer, inflammatory, and autoimmune diseases.

During the phase I studies, assessment of the maximum tolerated dose (MTD), after dosing range up to 100 mg twice daily, showed that the MTD was 75 mg twice daily based on dose-limiting toxicities (DLT) [25]. The pharmacodynamics evaluation by inhibition of p-AKT showed that inhibition of this mediator was not dose-dependent; it was maximal when duvelisib (**3**) was administered 25 mg twice daily. The use of duvelisib administered 25 mg twice daily advanced for the phase II and III studies [25]. Following administration of the duvelisib (**3**), the phosphorylated-AKT (p-AKT) levels were decreased. The p-AKT is a downstream marker of PI3K/AKT/mTOR pathway signaling. It is important to note that the administration of the duvelisib (**3**) leads to reduced levels of some chemokines, cytokines, and matrix metalloproteinases [25,26,27]. The reduction of several serum factors is consistent with alterations in the tumor microenvironment [25,26,27].

Duvelisib (**3**) is rapidly absorbed, with a maximum concentration after 1–3 h [25]. Following the administration of 25 mg of duvelisib (**3**), the absolute bioavailability in healthy volunteers is 42% [28]. Duvelisib (**3**) presents a volume of distribution of 28.5 L which is consistent with distribution to peripheral tissues. It is eliminated with a half-life of 4.7 h [28]. Duvelisib (**3**) is primarily metabolized by cytochrome P450 CYP3A4. The concomitant use with a strong inhibitor of CYP3A4 can increase the risk of toxic effects [4,28].

As expected, the most common adverse events in pretreated patients were the occurrence of opportunistic infections and cytopenias, with some cases resulting in fatal events [25,26,27,29]. Duvelisib (**3**) showed clinical efficacy and acceptable safety for the treatment of heavily pretreated patients with hematological malignancies, showing an overall response rate (ORR) higher than 50% [25,26,27,29].

In the phase III study for the relapsed or refractory CLL/SLL (NCT02004522) [6], the efficacy and safety of duvelisib (**3**) was compared with ofatumumab, an approved monoclonal antibody. Duvelisib (**3**) monotherapy showed a significant improvement of the ORR when compared with ofatumumab, resulting in the approval of this drug as an additional option for treatment of relapsed or refractory CLL/SLL [5].

Combinations of the duvelisib (**3**) with other approved drugs are under clinical studies for the treatment of cancer. Duvelisib (**3**) is being tested in combination with romidepsin or bortezomib against relapsed/refractory T-cell lymphomas (NCT02783625) [30]. Duvelisib (**3**) is also being combined with venetoclax for the treatment of relapsed or refractory CLL or SLL (NCT03534323) [31].

The clinical trials for the treatment of inflammatory and autoimmune diseases were both phase II studies, where the safety and efficacy of duvelisib (**3**) was tested for the treatment of asthma (NCT01653756) [32] and moderate to severe rheumatoid arthritis (NCT01851707) [33], respectively.

### 2.5. Syntheses

**Scheme 1:**
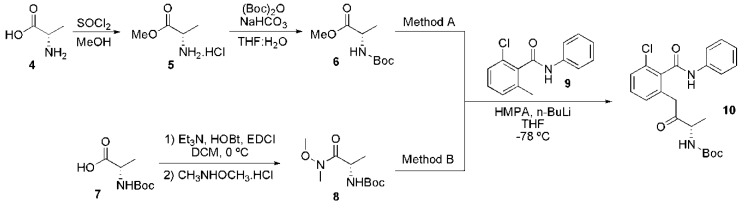
Methods for preparing the key intermediary, *tert*-butyl (*S*)-4-(3-chloro-2-(phenylcarbamoyl)phenyl)-3-oxobutan-2-ylcarbamate (**10**).

Duvelisib (**3**) can be obtained by two synthetic routes [34,35]. These routes differ in preparing the key intermediate, *tert*-butyl (*S*)-4-(3-chloro-2-(phenylcarbamoyl)phenyl)-3-oxobutan-2-ylcarbamate (**10**), from two different methods (Scheme 1).

In method A, the (S)-2-aminopropanoic acid (**4**) reacted with thionyl chloride and anhydrous methanol to form (*S*)-methyl 2-aminopropanoate hydrochloride (**5**). The amine group on **5** was protected by its reaction with di-*tert*-butyl dicarbonate. The protected intermediate **6** was then coupled with the carbanion derived from 2-chloro-6-methyl-*N*-phenylbenzamide (**9**), giving the key intermediate **10**. In method B, *N*,*O*-dimethylhydroxylamine hydrochloride was added to a mixture of (*S*)-2-(*tert*-butoxycarbonylamino)propanoic acid **7**, triethylamine, HOBt, EDCI in dichloromethane for the preparation of (*S*)-*tert*-butyl-(methoxy(methyl)amino)-1-oxopropan-2-ylcarbamate (**8**), which, after reaction with the carbanion of **9**, furnished the same key intermediate **10**.

**Scheme 2:**
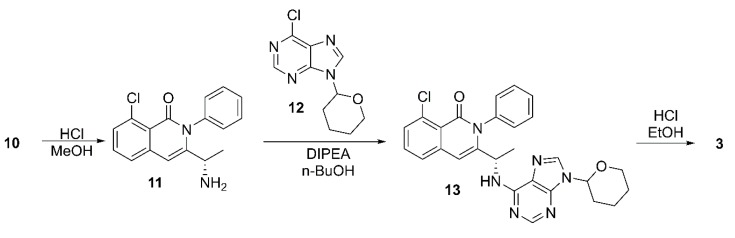
Preparation of duvelisib (**3**) from *tert*-butyl (*S*)-4-(3-chloro-2-(phenylcarbamoyl)phenyl)-3-oxobutan-2-ylcarbamate (**10**).

Treatment of the key intermediate **10** with hydrochloric acid in MeOH resulted in deprotection of the amine group and cyclization to form the isoquinolinone derivative **11** (Scheme 2). Next, the aromatic nucleophilic substitution reaction between **11** and 6-chloro-9-(tetrahydro-2H-pyran-2-yl)-9H-purine (**12**) was carried out, yielding 8-chloro-2-phenyl-3-((*1S*)-1-(9-(tetrahydro-*2H*-pyran-2-yl)-*9H*-purin-6-ylamino)ethyl) isoquinolin-1(*2H*)-one (**13**). Finally, compound **13** was treated with hydrochloric acid in ethanol to remove the THP protecting group, resulting in the formation of the desired compound, duvelisib (**3**).

## 3. Perspectives

The pharmacological profile of duvelisib (**3**) showed the safety and efficacy of this drug in the treatment of several hematological malignancies, indicating that it can be further approved for other uses beyond CLL, SLL, and FL. The use of duvelisib (**3**) for inflammatory and autoimmune diseases will be contested by the adverse events that occurred during the clinical trials, but the use of this drug for these applications probably will depend on the clinical benefit. The usefulness of the modulation of PI3K can be highlighted by the efforts on the discovery and development of novel PI3Kδ, PI3Kγ, and PI3Kδ/γ for the treatment of cancer, inflammatory, and autoimmune diseases [22,23].

## Figures and Tables

**Table 1:** Inhibitory profile of idelalisib (**1**), copanlisib (**2**), and duvelisib (**3**) against each PI3K isoform (IC_50_ values in nM).

Drug	PI3Kα	PI3Kβ	PI3Kδ	PI3Kγ	Reference
Idelalisib (**1**)	8600	4000	19	2100	[13]
Copanlisib (**2**)	0.5	3.7	6.4	0.7	[3]
Duvelisib (**3**)	1602	85	2.5	27	[15]

## References

[1] Markham A. (2014). Idelalisib: First global approval. Drugs.

[2] Markham A. (2017). Copanlisib: First Global Approval. Drugs.

[3] Scott W.J., Hentemann M.F., Rowley R.B., Bull C.O., Jenkins S., Bullion A.M., Johnson J., Redman A., Robbins A.H., Esler W. (2016). Discovery and SAR of novel 2,3-dihydroimidazo[1,2-c]quinazoline PI3K inhibitors: Identification of copanlisib (BAY 80-6946). ChemMedChem.

[4] Blair H.A. (2018). Duvelisib: First global approval. Drugs.

[5] Flinn I.W., Hillmen P., Montillo M., Nagy Z., Illes A., Etienne G., Delgado J., Kuss B.J., Tam C.S., Gasztonyi Z. (2018). The phase 3 DUO trial: Duvelisib vs ofatumumab in relapsed and refractory CLL/SLL. Blood.

[6] A Phase 3 Study of Duvelisib Versus Ofatumumab in Patients with Relapsed or Refractory CLL/SLL (DUO). https://clinicaltrials.gov/ct2/show/NCT02004522.

[7] Study of Duvelisib in Combination with Rituximab vs Rituximab in Subjects with Previously Treated Follicular Lymphoma (DYNAMO + R). https://clinicaltrials.gov/ct2/show/NCT02204982.

[8] Walker E.H., Perisic O., Ried C., Stephens L., Williams R.L. (1999). Structural insights into phosphoinositide 3-kinase catalysis and signalling. Nature.

[9] Hennessy B.T., Smith D.L., Ram P.T., Lu Y., Mills G.B. (2005). Exploiting the PI3K/AKT pathway for cancer drug discovery. Nat. Rev. Drug Discov..

[10] Liu P., Cheng H., Roberts T.M., Zhao J.J. (2009). Targeting the phosphoinositide 3-kinase pathway in cancer. Nat. Rev. Drug Discov..

[11] Thorpe L.M., Yuzugullu H., Zhao J.J. (2015). PI3K in cancer: Divergent roles of isoforms, modes of activation and therapeutic targeting. Nat. Rev. Cancer.

[12] Garces A.E., Stocks M.J. (2018). Class 1 PI3K clinical candidates and recent inhibitor design strategies: A medicinal chemistry perspective. J. Med. Chem..

[13] Somoza J.R., Koditek D., Villasenor A.G., Novikov N., Wong M.H., Liclican A., Xing W., Lagpacan L., Wang R., Schultz B.E. (2015). Structural, biochemical, and biophysical characterization of idelalisib binding to phosphoinositide 3-kinase delta. J. Biol. Chem..

[14] Miller M.S., Thompson P.E., Gabelli S.B. (2019). Structural determinants of isoform selectivity in PI3K inhibitors. Biomolecules.

[15] Winkler D.G., Faia K.L., DiNitto J.P., Ali J.A., White K.F., Brophy E.E., Pink M.M., Proctor J.L., Lussier J., Martin C.M. (2013). PI3K-delta and PI3K-gamma inhibition by IPI-145 abrogates immune responses and suppresses activity in autoimmune and inflammatory disease models. Chem. Biol..

[16] Walker E.H., Pacold M.E., Perisic O., Stephens L., Hawkins P.T., Wymann M.P., Williams R.L. (2000). Structural determinants of phosphoinositide 3-kinase inhibition by wortmannin, LY294002, quercetin, myricetin, and staurosporine. Mol. Cell..

[17] Huang C.H., Mandelker D., Schmidt-Kittler O., Samuels Y., Velculescu V.E., Kinzler K.W., Vogelstein B., Gabelli S.B., Amzel L.M. (2007). The structure of a human p110alpha/p85alpha complex elucidates the effects of oncogenic PI3Kalpha mutations. Science.

[18] Williams R., Berndt A., Miller S., Hon W.C., Zhang X. (2009). Form and flexibility in phosphoinositide 3-kinases. Biochem. Soc. Trans..

[19] Traxler P., Furet P. (1999). Strategies toward the design of novel and selective protein tyrosine kinase inhibitors. Pharmacol. Ther..

[20] Berndt A., Miller S., Williams O., Le D.D., Houseman B.T., Pacold J.I., Gorrec F., Hon W.C., Liu Y., Rommel C. (2010). The p110 delta structure: Mechanisms for selectivity and potency of new PI(3)K inhibitors. Nat. Chem. Biol..

[21] Katso R., Okkenhaug K., Ahmadi K., White S., Timms J., Waterfield M.D. (2001). Cellular function of phosphoinositide 3-kinases: Implications for development, homeostasis, and cancer. Annu. Rev. Cell Dev. Biol..

[22] Cushing T.D., Metz D.P., Whittington D.A., McGee L.R. (2012). PI3Kdelta and PI3Kgamma as targets for autoimmune and inflammatory diseases. J. Med. Chem..

[23] Perry M.W.D., Abdulai R., Mogemark M., Petersen J., Thomas M.J., Valastro B., Eriksson A.W. (2018). Evolution of PI3Kgamma and delta inhibitors for inflammatory and autoimmune diseases. J. Med. Chem..

[24] Ali A.Y., Wu X., Eissa N., Hou S., Ghia J.E., Murooka T.T., Banerji V., Johnston J.B., Lin F., Gibson S.B. (2018). Distinct roles for phosphoinositide 3-kinases gamma and delta in malignant B cell migration. Leukemia.

[25] Flinn I.W., O’Brien S., Kahl B., Patel M., Oki Y., Foss F.F., Porcu P., Jones J., Burger J.A., Jain N. (2018). Duvelisib, a novel oral dual inhibitor of PI3K-delta,gamma, is clinically active in advanced hematologic malignancies. Blood.

[26] O’Brien S., Patel M., Kahl B.S., Horwitz S.M., Foss F.M., Porcu P., Jones J., Burger J., Jain N., Allen K. (2018). Duvelisib, an oral dual PI3K-delta,gamma inhibitor, shows clinical and pharmacodynamic activity in chronic lymphocytic leukemia and small lymphocytic lymphoma in a phase 1 study. Am. J. Hematol..

[27] Horwitz S.M., Koch R., Porcu P., Oki Y., Moskowitz A., Perez M., Myskowski P., Officer A., Jaffe J.D., Morrow S.N. (2018). Activity of the PI3K-delta,gamma inhibitor duvelisib in a phase 1 trial and preclinical models of T-cell lymphoma. Blood.

[28] Verastem Inc. (2018). Copiktra (Duvelisib), Capsules for Oral Use: US Prescribing Information. https://www.accessdata.fda.gov/drugsatfda_docs/label/2018/211155s000lbl.pdf.

[29] Flinn I.W., Patel M., Oki Y., Horwitz S., Foss F.F., Allen K., Douglas M., Stern H., Sweeney J., Kharidia J. (2018). Duvelisib, an oral dual PI3K-delta, gamma inhibitor, shows clinical activity in indolent non-Hodgkin lymphoma in a phase 1 study. Am. J. Hematol..

[30] Trial of Duvelisib in Combination with Either Romidepsin or Bortezomib in Relapsed/Refractory T-Cell Lymphomas. https://clinicaltrials.gov/ct2/show/NCT02783625.

[31] Duvelisib and Venetoclax in Relapsed or Refractory CLL or SLL. https://clinicaltrials.gov/ct2/show/NCT03534323.

[32] A Phase 2a, Efficacy and Safety Study of Duvelisib in Mild Asthmatic Subjects. https://clinicaltrials.gov/ct2/show/NCT01653756.

[33] A Double-Blind Study Evaluating Duvelisib in Subjects with Moderate to Severe Rheumatoid Arthritis and an Inadequate Response to Methotrexate Alone (ASPIRA). https://clinicaltrials.gov/ct2/show/NCT01851707.

[34] Ren P., Liu Y., Wilson T.E., Li L., Chan K., Rommel C. (2009). Certain Chemical Entities, Compositions and Methods.

[35] Ren P., Liu Y., Wilson T.E., Li L., Chan K., Rommel C. (2011). Certain Chemical Entities, Compositions and Methods.

